# ULK1 gene polymorphisms and severe tuberculosis in the Chinese Han population: a case-control study

**DOI:** 10.3389/fmed.2025.1635313

**Published:** 2025-09-09

**Authors:** Juan Zhang, Jian-Qing He

**Affiliations:** ^1^Intensive Care Unit, Deyang People’s Hospital, Deyang, Sichuan, China; ^2^Department of Respiratory and Critical Care Medicine, West China Hospital of Sichuan University, Chengdu, Sichuan, China

**Keywords:** severe tuberculosis, genetic polymorphism, clinical phenotype, ULK1, Chinese Han population

## Abstract

**Objectives:**

Polymorphisms in the uncoordinated 51-like kinase 1 (ULK1) gene are associated with susceptibility to multiple diseases, including neurodegenerative disorders and specific cancer types. In tuberculosis (TB) research, autophagy is recognized as an essential host mechanism against *Mycobacterium tuberculosis* (Mtb) infection. Consequently, functional variations in the ULK1 gene may affect autophagic efficiency, influencing the host immune response to Mtb and altering the severity of TB. This study aimed to investigate the association between ULK1 gene polymorphisms and severe TB within a Chinese Han population using a case–control study design.

**Methods:**

A case–control study was conducted, with patients diagnosed with mild TB as controls and those diagnosed with severe TB as cases. Peripheral blood samples were collected from all participants for genomic DNA extraction. Four tag single nucleotide polymorphisms (SNPs) within the ULK1 gene (rs9481, rs7138581, rs11616018, and rs1134574) were selected based on genotype data from the Han Chinese population in Beijing. The association between these SNPs and TB severity was analyzed. Additionally, clinical phenotype analysis was conducted for the loci associated with TB severity.

**Results:**

The minor allele G of the ULK1 gene SNP rs1134574 (A > G) was significantly associated with an increased risk of severe TB (OR^a^ = 23.499, 95% CI = 7.339–75.249, P^a^ < 0.0001). However, no statistically significant difference was observed in genotype frequencies or genetic models at this locus between severe and mild TB groups. Clinical phenotype analysis identified 995 patients with the AA genotype, 136 patients with the AG genotype, and 6 patients with the GG genotype at rs1134574. Significant differences were observed among these genotypes regarding the proportion of patients experiencing night sweats (*p* = 0.045) and the percentage of neutrophils (*p* = 0.046).

**Conclusion:**

The polymorphism rs1134574 of the ULK1 gene is significantly associated with severe TB, and clinical phenotype variations exist among different genotypes at this locus. These findings suggest a potential correlation between ULK1 gene polymorphisms and the incidence of severe TB within the Chinese Han population.

## Introduction

Tuberculosis (TB), caused by *Mycobacterium tuberculosis* (Mtb), is a chronic infectious disease representing a significant global public health threat (1). Severe TB is defined by progression to advanced disease stages, frequently resulting in extensive tissue damage, organ failure, and elevated mortality rates (2–4). While the standard anti-TB treatment regimen effectively cures the majority of patients, those with severe TB often encounter increased risks due to suboptimal treatment outcomes (5).

The influence of genetic factors on TB susceptibility and disease progression has recently become a prominent research focus. Several studies indicate that host genetic variations may significantly influence immune responses to Mtb, as well as the development and severity of TB (6–8). Among these genetic variations, the role of autophagy-related genes is particularly significant. Autophagy is an intracellular process responsible for the degradation and recycling of cellular components, essential for maintaining cellular homeostasis and clearing invasive pathogens (9). The protein kinase complex encoded by the uncoordinated 51-like kinase 1 (ULK1) gene plays a central role in initiating autophagosome formation (10, 11). Therefore, functional polymorphisms in the ULK1 gene may influence autophagy efficiency, consequently affecting the host’s capacity to clear Mtb.

Recent research has started to explore the association between ULK1 gene polymorphisms and various diseases (12, 13). However, there is a notable lack of studies specifically addressing this relationship in TB, particularly regarding severe disease forms. Given this knowledge gap, investigating the potential association between ULK1 gene polymorphisms and severe TB is essential to enhance the understanding of TB pathophysiology and clinical heterogeneity. This study aims not only to identify potential genetic risk factors for severe TB but also to uncover novel therapeutic targets. Such findings may facilitate the development of more precise clinical strategies for prevention, diagnosis, and treatment.

## Materials and methods

### Ethics approval

This study was approved by the Ethics Committee of West China Hospital, Sichuan University (approval no. 932 [2019]). The study objectives and methods were thoroughly explained to all participants, each of whom provided informed consent prior to participation. For minors (individuals aged <18 years), legal guardians provided consent on their behalf. In cases where participants lacked the capacity to consent due to illness, legal guardians signed the consent form.

### Study design

The study was structured into three distinct components. Firstly, clinical data for patients were collected from the electronic medical record system, encompassing variables including age, gender, living environment, smoking and drinking habits, clinical symptoms, laboratory test results, sputum smear status, retreatment status, and multidrug-resistant tuberculosis (MDR-TB) status. Subsequently, a comparative analysis of clinical characteristics between patients with mild and severe TB was conducted. A molecular genetic susceptibility analysis for severe TB was then performed. The study comprised 558 patients with severe TB (case group) and 579 patients with mild TB (control group). DNA was extracted from peripheral blood samples obtained from all patients. Tag SNPs were selected based on genotype data from the Han Chinese population in Beijing, resulting in the selection of four specific ULK1 gene loci (rs9481, rs7138581, rs11616018, and rs1134574) for investigation in relation to TB severity. DNA extraction was conducted using the Tiangen Biotech DNA Extraction Kit (Beijing, China), and the concentration and purity of DNA in all samples were subsequently measured.

High-throughput SNP genotyping technology was employed to genotype the selected SNP sites. The allele frequency and genotype frequency distributions between mild and severe TB cases were compared to identify statistical differences. Four genetic models were analyzed: allelic, additive, dominant, and recessive. Finally, SNP loci identified as significantly associated with TB severity in the association analysis underwent clinical phenotype analysis, comparing differences in clinical symptoms, laboratory results, and other factors among different genotypes.

### Study population

From January 2013 to December 2020, a total of 558 patients with severe TB and 579 patients with mild TB, aged 15–45 years and of Han ethnicity, were admitted to West China Hospital, Sichuan University. All included patients were unrelated individuals. The TB cases in this study were both clinically diagnosed and bacteriologically confirmed, adhering to relevant diagnostic criteria guidelines (14, 15). Patients with autoimmune deficiency syndrome, hepatitis B, diabetes, or history of organ transplantation were excluded.

### Definition of severe TB

Currently, universally accepted criteria for severe TB remain lacking. Based on a comprehensive literature review and guidelines from the World Health Organization (WHO), this study defines severe TB as meeting at least one of the following criteria (14–19):

Multi-system involvement: TB affecting two or more organ systems (e.g., pulmonary disease with concurrent meningeal or miliary dissemination).

Critical neurological involvement: Tuberculous meningitis, regardless of stage.

Disseminated disease: Hematogenous disseminated pulmonary TB.

Radiological severity: Lung cavities exceeding 4 cm in diameter, or bilateral pulmonary lesions involving >2/3 of the lung fields.

### Tag SNP selection, DNA extraction, and genotyping

In the selection of tag SNPs, data from the Han Chinese population in Beijing, China were utilized. The selected tag SNPs adhered to a minimum allele frequency (MAF) of 5% and an r^2^ value ranging from 0.8 to 1.0. Four specific tag SNP sites within the ULK1 gene, rs9481, rs7138581, rs11616018, and rs1134574, were included in this study. DNA extraction was performed using the Tiangen DNA Extraction Kit (Beijing, China) on 200 μL peripheral blood samples collected from all TB patients. All extracted DNA samples underwent assessment for concentration and purity, with samples considered qualified if their concentration exceeded 15 ng/μL and purity ranged from 1.8 to 2.0. Qualified samples were subsequently stored at −80°C. SNPscan™ multiplex SNP genotyping technology employed probes corresponding to distinct sequences at various SNP sites, thus enabling differentiation between alleles through detection of specific fluorescent signals.

### Sample size calculation

Sample size was calculated using PASS 2023 software, with parameters set at *α* = 0.05 and statistical power (1-*β*) = 0.8. The statistical power for selected SNPs loci across various genetic models was subsequently evaluated using PASS 2023 software.

### Statistical analysis

In comparing baseline data between mild and severe TB groups, as well as clinical phenotypic analyses of TB severity-associated loci, quantitative data are presented as mean ± standard deviation (SD), while qualitative data are expressed as proportions. The statistical significance of quantitative data was determined using t-tests or Mann–Whitney U tests, while chi-square tests were employed to compare proportions for qualitative data. Additionally, chi-square tests were used to ascertain statistically significant differences in allele and genotype frequency distributions between mild and severe TB cases. Four genetic models, allelic, additive, dominant, and recessive, were employed in the gene model analysis. Binary logistic regression was utilized to calculate associations between SNPs and TB severity, adjusting for confounding factors such as age and gender. To ensure the representativeness of the study sample, chi-square tests were conducted to confirm adherence of genotype frequencies to Hardy–Weinberg equilibrium (HWE). Finally, the online analysis tool SHEsis was employed to investigate linkage disequilibrium (LD) relationships among genetic markers and to assess haplotype composition (20).

## Results

### Characteristics of the study participants

**Figure 1:**
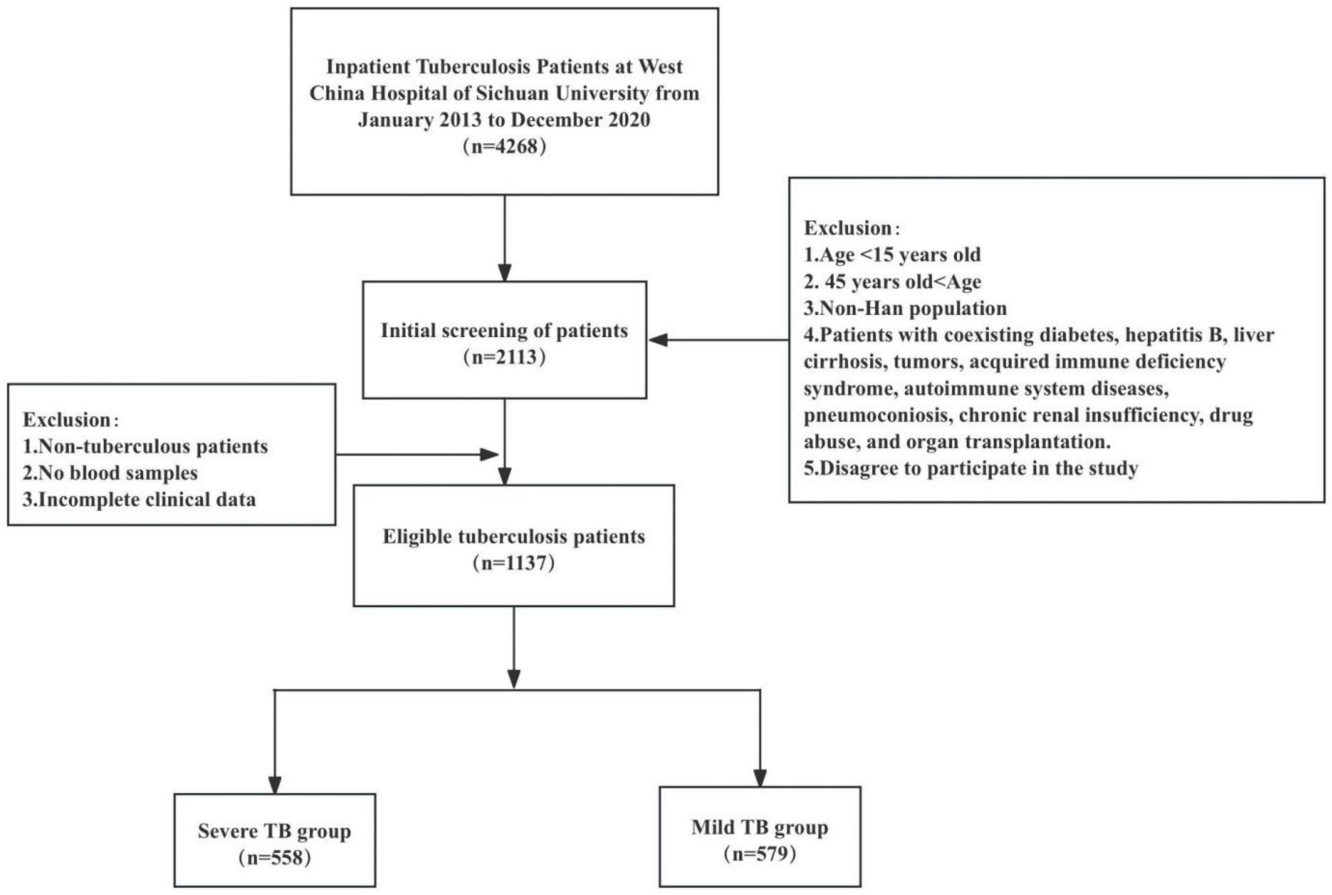
The screening flow of patients.

In this study, 558 patients with severe TB and 579 patients with mild TB were observed. The patient screening process is shown in Figure 1. The mean age of patients in the severe TB group was 26.79 ± 8.17 years, slightly lower than 29.01 ± 8.20 years in the mild TB group. Smoking prevalence was notably higher in the severe TB group (24.6%) compared to the mild TB group (18.7%). Coughing and expectoration were the most common symptoms reported in both groups, affecting 47.5% of severe TB patients and 50.1% of mild TB patients. This was closely followed by fever, present in 39.1% of severe TB patients compared to 20.7% in the mild TB group. A higher proportion of patients in the severe TB group had a history of previous TB treatment (25.1%) compared to the mild TB group (14.5%). Additionally, positive sputum smear results were more prevalent in the severe TB group (29.6%) than in the mild TB group (19.0%). The prevalence of MDR-TB was slightly elevated in the severe TB group (3.4%) compared with the mild TB group (2.6%), as summarized in Table 1.

**Table 1:** Clinical characteristics of severe and mild TB.

Features	Severe TB	Mild TB	*p*
Age, years (mean ± SD)	26.79 ± 8.166	29.01 ± 8.199	<0.001
Sex, *N* (%)			
Females	308 (55.2)	298 (51.5)	0.208
Males	250 (44.8)	281 (48.5)	
Living environment, *N* (%)			0.901
Countryside	421 (75.4)	435 (75.1)	
Towns	137 (24.6)	144 (24.9)	
Smoking, *N* (%)			
Yes	137 (24.6)	108 (18.7)	0.016
No	421 (75.4)	471 (81.3)	
Drinking, *N* (%)			
Yes	70 (12.5)	53 (9.2)	0.067
No	488 (87.5)	526 (90.8)	
Symptoms, *N* (%)			
Headache	74 (13.3)	2 (0.3)	<0.001
Mass	46 (7.9)	43 (7.7)	0.881
Joint pain	3 (0.5)	1 (0.2)	0.324
Fatigue and anorexia	43 (7.7)	24 (4.1)	0.012
Hemoptysis	43 (7.7)	101 (17.4)	<0.001
Cough and expectoration	265 (47.5)	290 (50.1)	0.382
Fever	218 (39.1)	120 (20.7)	<0.001
Tidal fever and night sweating	43 (7.7)	38 (6.6)	0.454
Shortness of breath	71 (12.7)	66 (11.4)	0.493
Chest tightness	14 (2.5)	27 (4.7)	0.055
Chest pain	44 (7.9)	93 (16.1)	<0.001
Nausea and vomiting	23 (4.1)	7 (1.2)	0.004
Abdominal pain	76 (13.6)	23 (4.0)	<0.001
Abdominal distension	66 (11.8)	19 (3.3)	<0.001
Diarrhea	20 (4.0)	1 (0.2)	0.002
Backache	13 (2.6)	10 (1.8)	0.346
Urinary tract irritation	5 (1.0)	6 (1.1)	0.930
Hematuria	1 (0.2)	1 (0.2)	0.927
Laboratory examinations (Mean ± SD)			
White blood cell count (*10^9/L)	7.33 ± 3.745	6.02 ± 2.557	<0.001
Percentage of lymphocytes (%)	16.79 ± 9.280	23.15 ± 9.573	<0.001
Percentage of neutrophils (%)	73.33 ± 11.659	65.71 ± 11.107	<0.001
Hemoglobin (g/L)	115.76 ± 23.526	128.34 ± 20.030	<0.001
Platelets (*10^9/L)	303.28 ± 141.991	248.84 ± 106.909	<0.001
Total bilirubin (μmol/L)	12.10 ± 19.548	13.56 ± 32.292	0.385
Direct bilirubin (μmol/L)	5.64 ± 4.349	5.61 ± 3.634	0.902
ALT (U/L)	39.19 ± 72.466	43.13 ± 87.475	0.501
AST (U/L)	36.45 ± 70.355	46.04 ± 119.748	0.152
Albumin (g/L)	35.02 ± 6.189	39.26 ± 5.380	<0.001
Creatinine (μmol/L)	58.36 ± 37.063	64.51 ± 32.836	0.10
Uric acid (μmol/L)	418.07 ± 187.548	415.43 ± 170.801	0.810
CRP (mg/L)	43.07 ± 44.774	23.93 ± 35.453	<0.001
PCT (ng/ml)	0.14 ± 0.796	0.10 ± 1.451	0.549
ESR (mm/h)	54.93 ± 35.245	39.18 ± 31.592	<0.001
Retreatment, *N* (%)			
Initial treatment	418 (74.9)	495 (85.5)	<0.001
Retreatment	140 (25.1)	84 (14.5)	
Sputum smear, *N* (%)			
Negative	393 (70.4)	469 (81.0)	<0.001
Positive	165 (29.6)	110 (19.0)	
MDT-TB, *N* (%)			
Yes	539 (96.6)	564 (97.4)	0.422
No	19 (3.4)	15 (2.6)	

TB, Tuberculosis; ALT, Alanine aminotransferase; AST, Aspartate aminotransferase; CRP, C-reactive protein; ESR, Erythrocyte sedimentation rate; PCT, Procalcitonin; MDR-TB, Multidrug resistant tuberculosis.

### Polymorphism in the ULK1 gene and TB severity

Four tag SNP loci within the ULK1 gene (rs9481, rs7138581, rs11616018, and rs1134574) were selected for analysis. The details of these SNPs are provided in Table 2. Logistic regression analysis indicated that the minor allele G of rs1134574 (A > G) significantly increased the risk of severe TB (OR = 23.034; 95% CI: 7.212–73.567; *p* < 0.0001). However, no significant correlation was found in the genotype frequency distributions (AA, AG, GG) or genetic model analyses (additive, dominant, and recessive models), with all *p*-values exceeding 0.05. After adjusting for age and gender as confounding factors in the logistic regression analysis, the minor allele G of rs1134574 remained significantly associated with increased severe TB risk (OR^a^ = 23.499; 95% CI: 7.339–75.249; *p* < 0.0001). Conversely, no significant associations were observed between the genotype frequency distributions of rs9481, rs7138581, or rs11616018 and severe TB in genetic model analyses, as presented in Table 3.

**Table 2:** The basic information of SNPs.

Gene	Chromosome	SNPs	SNPs location	Functional Consequence	Alleles	MAF(CHB) 1000genomes
*ULK1*	12	rs9481	131,922,544	3 Prime UTR Variant	A > G	0.08
*ULK1*	12	rs7138581	131,922,121	3 Prime UTR	G > C	0.11
*ULK1*	12	rs11616018	131,912,058	Synonymous Variant	C > T	0.337
*ULK1*	12	rs1134574	131,897,456	Intron Variant	A > G	0.063

**Table 3:** Haplotype analysis of tuberculosis severity.

Haplotype	Severe TB *N* (%)	Mild TB *N* (%)	OR^a^ (95%CI)	P^a^
GCCG	69 (6.2)	71 (6.1%)	1.02 (0.726–1.439)	0.900
GCTA	99 (8.9)	134 (11.6)	0.746 (0.567–0.981)	0.036
GGCA	57 (5.1)	74 (6.4)	0.786 (0.551–1.122)	0.184
GGTA	277 (24.8)	254 (22.0)	1.18 (0.971–1.435)	0.096

OR, odds ratio; CI, confidence interval; a, adjusted for age and gender.

### HWE test and haplotype analysis

The HWE test was performed on both mild and severe TB groups, with *p*-values greater than 0.05 in all instances, indicating compliance with HWE and confirming the representativeness of the selected study population (Table 4).

**Table 4:** Hardy-Weinberg equilibrium results for all SNPs in cases and controls.

Frequency	Severe TB group Genotype			Mild TB group Genotype		
	AA	AG	GG	AA	AG	GG
Actual frequency	486	69	3	509	67	3
Theoretical frequency	485.5	69.9	2.5	508	68	2
χ^2^	0.104			0.517		
*p*	0.949			0.772		

SNP, single nucleotide polymorphism; TB, tuberculosis.

**Figure 2:**
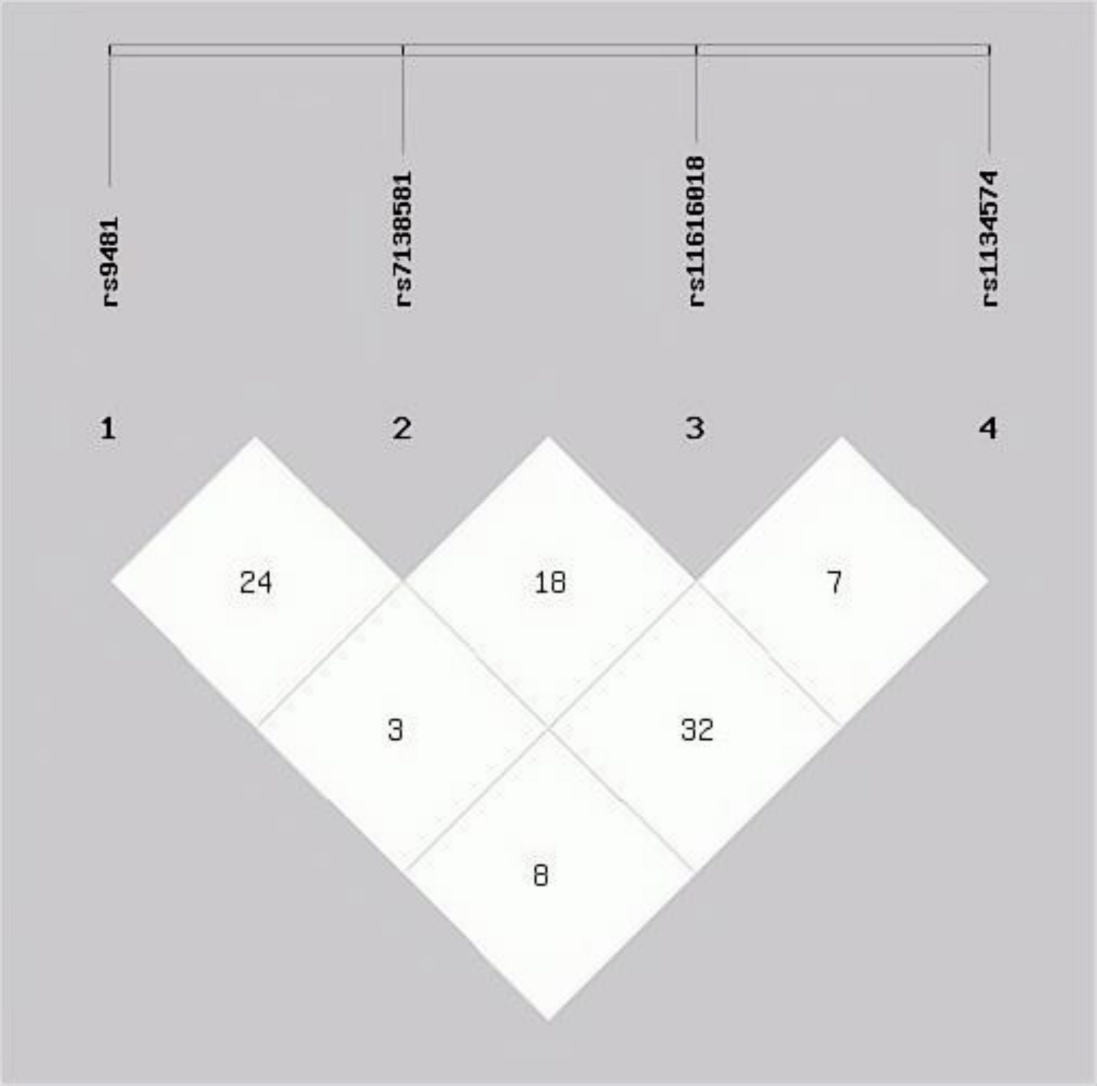
Linkage disequilibrium tests of all four tagSNPs displayed in the form of r2.

LD analysis among the four selected tag SNPs in the ULK1 gene is depicted in Figure 2. Haplotype analysis was conducted using the SHEsis software platform, excluding haplotypes with frequencies below 0.03. Four major haplotypes (GCCG, GCTA, GGCA, and GGTA) were identified when examining associations between ULK1 SNPs and TB severity. Notably, the GCTA haplotype was significantly associated with a decreased risk of severe TB (OR^a^ = 0.746; 95% CI: 0.567–0.981; Pa = 0.036). Additional detailed results are presented in Table 5.

**Table 5:** Polymorphism of ULK1 gene and the severity of tuberculosis.

SNPs	Genetic models	Allele/Genotype	Severe TB *N* (%)	Mild TB *N* (%)	OR (95%CI)	*p*	OR^a^ (95%CI)	P^a^
rs948 (A > G)		A	607 (54.4)	618 (53.4)	1	References	1	Reference
		G	509 (45.6)	540 (46.6)	0.960 (0.814–1.132)	0.625	0.964 (0.816–1.13)	0.666
		AA	160 (28.7)	159 (27.5)	1	References	1	Reference
		AG	287 (51.4)	300 (51.8)	0.951 (0.724–1.249)	0.716	0.961 (0.730–1.26)	0.777
		GG	111 (19.9)	120 (20.7)	0.919 (0.655–1.290)	0.626	0.927 (0.658–1.30)	0.663
	Additive	2GG + AGvsAA			0.958 (0.810–1.134)	0.618	0.963 (0.812–1.14)	0.660
	Dominant	AG + GGvsAA			0.942 (0.727–1.220)	0.649	0.951 (0.732–1.23)	0.708
	Recessive	AAvsAG + GG			0.989 (0.906–1.079)	0.803	0.992 (0.909–1.08)	0.861
rs7138581 (G > C)		G	943 (84.5)	949 (82.0)	1	References	1	Reference
		C	173 (15.5)	209 (18.0)	0.833 (0.668–1.039)	0.105	0.844 (0.675–1.05)	0.135
		GG	393 (70.4)	387 (66.8)	1	References	1	Reference
		GC	157 (28.1)	175 (30.2)	0.883 (0.683–1.142)	0.345	0.891 (0.687–1.15)	0.384
		CC	8 (1.4)	17 (2.9)	0.463 (0.198–1.086)	0.077	0.487 (0.206–1.15)	0.101
	Additive	2CC + GCvsGG			0.826 (0.659–1.035)	0.097	0.837 (0.666–1.051)	0.126
	Dominant	GC + CCvsGG			0.846 (0.658–1.088)	0.192	0.856 (0.665–1.10)	0.230
	Recessive	GGvsGC + CC			0.959 (0.880–1.045)	0.335	0.962 (0.882–1.04)	0.375
rs11616018 (C > T)		C	719 (64.4)	748 (64.6)	1	References	1	Reference
		T	397 (35.6)	410 (35.4)	1.007 (0.848–1.196)	0.993	1.016 (0.854–1.20)	0.858
		CC	236 (42.3)	235 (40.6)	1	References	1	Reference
		CT	247 (44.3)	278 (48.0)	0.885 (0.690–1.135)	0.335	0.895 (0.696–1.15)	0.389
		TT	75 (11.4)	66 (11.4)	1.132 (0.776–1.650)	0.520	1.148 (0.785–1.67)	0.478
	Additive	2TT + CTvsCC			1.007 (0.848–1.197)	0.933	1.016 (0.854–1.21)	0.857
	Dominant	TT + CTvsCC			0.932 (0.736–1.180)	0.559	0.944 (0.744–1.19)	0.634
	Recessive	TTvsCT + CC			0.955 (0.880–1.037)	0.273	0.959 (0.882–1.04)	0.318
rs1134574 (A > G)		A	1,053 (94.4)	1,155 (99.7)	1	References	1	Reference
		G	63 (5.6)	3 (0.3)	23.034 (7.212–73.567)	<0.0001	23.499 (7.339–75.249)	<0.0001
		AA	486 (87.1)	509 (87.9)	1	References	1	Reference
		AG	69 (12.4)	67 (11.6)	1.079 (0.754–1.544)	0.679	1.073 (0.747–1.541)	0.703
		GG	3 (0.5)	3 (0.5)	1.047 (0.210–5.214)	0.955	0.985 (0.196–4.959)	0.986
	Additive	2GG + AGvsAA			1.070 (0.769–1.489)	0.690	1.060 (0.759–1.479)	0.734
	Dominant	GG + AGvsAA			1.077 (0.758–1.531)	0.678	1.069 (0.750–1.525)	0.713
	Recessive	GGvsAG + AA			1.026 (0.910–1.156)	0.678	1.024 (0.907–1.155)	0.704

SNPs, single nucleotide polymorphisms; OR, odds ratio; CI, confidence interval; a, adjusted for age and gender.

### Sample size calculation and sample efficacy analysis

**Figure 3:**
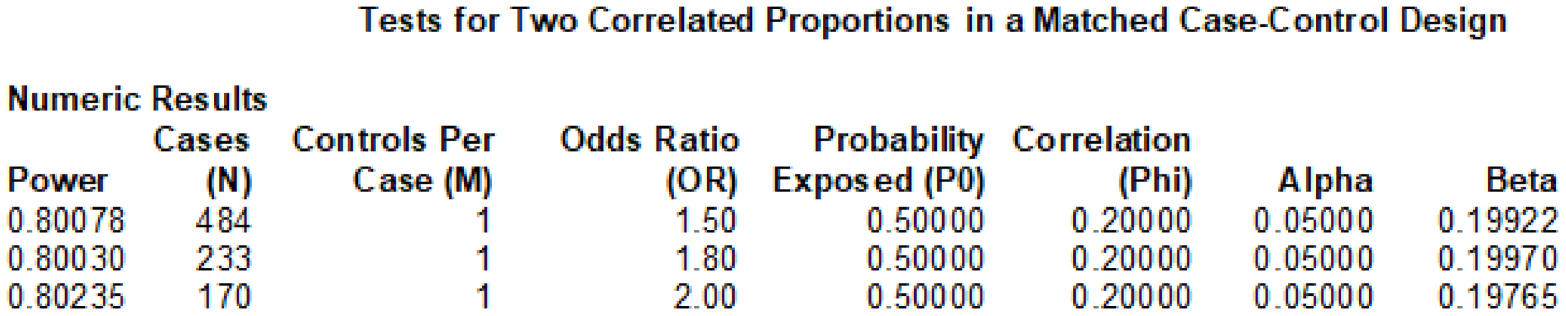
Sample size calculation.

With a set significance level (*α* = 0.05) and statistical power (1 − *β* = 0.8), sample size calculations were performed using PASS 2023 software for odds ratios (ORs) of 1.5, 1.8, and 2.0, resulting in required case-group sample sizes of 484, 233, and 170, respectively (Figure 3). Additionally, we conducted sample power analyses for the four selected SNP loci across various genetic models using PASS 2023 software; results are presented in Table 6.

**Table 6:** Study sample efficacy analysis.

Gene	SNPs	MAF (%)	Genetic models	OR = 1.5	OR = 2	OR = 2.5	OR = 3
*ULK1*	rs9481	0.08	Allele	0.831	0.998	1.000	1.000
			Dominant	0.808	0.999	1.000	1.000
			Recessive	0.198	0.448	0.669	0.818
			Additive	0.860	0.999	1.000	1.000
*ULK1*	rs7138581	0.11	Allele	0.914	0.999	1.000	1.000
			Dominant	0.893	0.999	1.000	1.000
			Recessive	0.276	0.633	0.858	0.953
			Additive	0.935	0.999	1.000	1.000
*ULK1*	rs11616018	0.337	Allele	0.996	1.000	1.000	1.000
			Dominant	0.995	1.000	1.000	1.000
			Recessive	0.900	0.999	1.000	1.000
			Additive	0.996	1.000	1.000	1.000
*ULK1*	rs1134574	0.063	Allele	0.752	0.994	0.999	1.000
			Dominant	0.732	0.992	0.999	1.000
			Recessive	0.163	0.348	0.532	0.680
			Additive	0.776	0.996	0.999	1.000

### Clinical phenotype analysis of TB severity-associated loci

In the previous correlation analysis, a significant association was identified between the ULK1 gene SNP rs1134574 and TB severity. Specifically, the AA genotype was observed in 995 patients, the AG genotype in 136 patients, and the GG genotype in only 6 patients. Clinical phenotype analyses were performed for these three genotypes. A statistically significant difference was observed among genotypes regarding the proportion of patients experiencing tidal fever and night sweats (*p* = 0.045). However, no statistically significant differences were found among the genotypes for other clinical symptoms. Additionally, neutrophil percentages differed significantly across the AA, AG, and GG genotypes (*p* = 0.046; Table 7). No statistically significant differences were identified among genotypes in terms of sputum bacterial load, ICU admission rates, proportion requiring retreatment, length of hospital stay, rates of concurrent bacterial and fungal infections, or incidence of shock.

**Table 7:** Clinical phenotypes of different genotypes of ULK1 gene rs1134574.

Features	Genotype AA *N* = 995	Genotype AG *N* = 136	Genotype GG *N* = 6	*p*
Symptoms, *N* (%)				
Headache	62 (6.2)	13 (9.6)	1 (16.7)	0.214
Obstruction of consciousness	10 (1.0)	0 (0.0)	0 (0.0)	0.487
Convulsions	2 (0.2)	0 (0.0)	0 (0.0)	0.867
Mass	74 (7.4)	15 (11.0)	0 (0.0)	0.266
Joint pain	4 (0.4)	0 (0.0)	0 (0.0)	0.751
Hoarseness	2 (0.2)	1 (0.7)	0 (0.0)	0.518
Fatigue and anorexia	56 (5.6)	11 (8.1)	0 (0.0)	0.431
Hemoptysis	131 (13.2)	12 (8.8)	1 (16.7)	0.345
Cough and expectoration	482 (48.4)	68 (50.0)	5 (83.3)	0.224
Fever	300 (30.2)	35 (25.7)	3 (50.0)	0.316
Tidal fever and night sweating	78 (7.8)	3 (2.2)	0 (0.0)	0.045
Shortness of breath	120 (12.1)	17 (12.5)	0 (0.0)	0.654
Chest tightness	33 (3.3)	8 (5.9)	0 (0.0)	0.288
Chest pain	115 (11.6)	21 (15.4)	1 (16.7)	0.402
Nausea and vomiting	28 (2.8)	2 (1.5)	0 (0.0)	0.605
Abdominal pain	88 (8.8)	10 (7.4)	1 (16.7)	0.665
Abdominal distension	72 (7.2)	13 (9.6)	0 (0.0)	0.492
Diarrhea	21 (2.3)	0 (0.0)	0 (0.0)	0.224
Backache	22 (2.4)	1 (0.8)	0 (0.0)	0.500
Urinary tract irritation	10 (1.1)	1 (0.8)	0 (0.0)	0.932
Hematuria	2 (0.2)	0 (0.2)	0 (0.2)	0.869
Laboratory examinations (Mean ± SD)				
White blood cell count (*10^9/L)	6.72 ± 3.327	6.21 ± 2.800	8.43 ± 2.438	0.117
Percentage of lymphocytes (%)	19.83 ± 9.838	21.33 ± 10.721	13.52 ± 3.971	0.094
Percentage of neutrophils (%)	69.65 ± 11.913	67.82 ± 12.630	78.63 ± 4.505	0.046
Hemoglobin (g/L)	122.32 ± 22.656	120.76 ± 23.190	118.80 ± 23.156	0.722
Platelets (*10^9/L)	276.83 ± 128.962	268.83 ± 126.628	292.67 ± 97.760	0.759
Total bilirubin (μmol/L)	12.34 ± 21.375	16.55 ± 50.996	9.38 ± 7.529	0.234
Direct bilirubin (μmol/L)	5.62 ± 4.136	5.64 ± 2.986	4.97 ± 1.305	0.922
ALT (U/L)	41.30 ± 82.518	40.63 ± 61.715	22.60 ± 25.046	0.872
AST (U/L)	42.42 ± 102.649	33.06 ± 54.138	15.20 ± 9.524	0.551
Albumin (g/L)	37.18 ± 6.051	37.03 ± 7.068	35.65 ± 3.871	0.810
Creatinine (μmol/L)	60.97 ± 31.496	64.71 ± 54.833	67.30 ± 25.587	0.494
Uric acid (μmol/L)	419.59 ± 178.032	398.93 ± 189.991	343.80 ± 73.333	0.313
CRP (mg/L)	26.28 ± 39.575	21.32 ± 34.630	39.42 ± 38.371	0.267
PCT (ng/ml)	0.10 ± 0.652	0.30 ± 2.910	0.04 ± 0.033	0.163
ESR (mm/h)	42.61 ± 36.195	37.22 ± 29.968	48.17 ± 41.412	0.235
Other indicators				
Bacterial load (−), *N* (%)	748 (75.2)	110 (80.9)	4 (66.7)	0.381
Bacterial load (+), *N* (%)	206 (20.7)	19 (14.0)	2 (33.3)	
Bacterial load (++), *N* (%)	28 (2.8)	3 (2.2)	0 (0.0)	
Bacterial load (+++), *N* (%)	13 (1.3)	4 (2.9)	0 (0.0)	
Retreatment, *N* (%)	197 (19.8)	24 (17.6)	3 (50.0)	0.146
MDR-TB, *N* (%)	33 (3.3)	1 (1.7)	0 (0.0)	0.231
Pulmonary cavity, *N* (%)	215 (21.6)	25 (18.4)	0 (0.0)	0.307
Co-bacterial infection, *N* (%)	207 (20.8)	19 (14.0)	0 (0.0)	0.082
Co-fungal infection, N(%)	23 (2.3)	1 (0.7)	0 (0.0)	0.456
Respiratory failure, *N* (%)	19 (1.9)	1 (0.7)	0 (0.0)	0.588
Sepsis, *N* (%)	2 (0.2)	0 (0.0)	0 (0.0)	0.867
Sepsis shock, *N* (%)	1 (0.1)	0 (0.0)	0 (0.0)	0.931
Invasive ventilator, *N* (%)	3 (0.3)	1 (0.7)	0 (0.0)	0.718
ICU, *N* (%)	13 (1.3)	2 (1.5)	0 (0.0)	0.949
Length of stay (mean ± SD)	14.74 ± 10.173	15.43 ± 9.880	18.83 ± 2.041	0.765

ALT, Alanine aminotransferase; AST, Aspartate aminotransferase; CRP, C-reactive protein; ESR, Erythrocyte sedimentation rate; PCT, Procalcitonin; MDR-TB, Multidrug resistant tuberculosis; ICU, Intensive care unit.

## Discussion

China ranks third among the 30 nations with the highest TB burden, contributing 7.1% of global cases (1). Despite a declining trend in TB incidence within China, the disease’s impact remains substantial. Although China’s TB mortality rate is relatively low compared with other countries, the absolute number of deaths remains high due to its vast population, posing significant public health challenges (21, 22). Consequently, additional efforts are necessary to achieve the WHO’s goal of “Ending the TB Epidemic”.

Patients with TB may progress to severe forms of the disease due to various factors, including delayed diagnosis, irregular treatment, emergence of drug-resistant strains, and host-related factors (2, 23, 24). These patients often rapidly develop respiratory failure and extrapulmonary organ dysfunction, potentially resulting in fatal outcomes. Severe TB is primarily characterized by marked clinical symptoms, extended treatment durations, high complexity of medical management, substantial healthcare costs, and elevated mortality and disability rates (24). The financial burden, societal impacts, psychological stress, and associated challenges make severe TB a critical issue for TB prevention and control.

Currently, there is no universally accepted definition or diagnostic criterion for severe TB, either nationally or internationally. Additionally, studies specifically addressing severe TB are limited, and existing literature lacks clear inclusion and exclusion criteria for defining this condition. Although numerous studies have confirmed genetic susceptibility to TB, research investigating genetic predisposition specifically for severe TB, particularly among the Chinese population, remains scarce (3, 25, 26). Given that host genetic SNPs can influence the onset, progression, and outcomes of TB, these genetic variations might affect not only disease susceptibility but also disease severity.

Currently, there is a scarcity of both domestic and international studies examining the association between host gene SNPs and TB severity. Existing research primarily focuses on the relationship between host gene SNPs and radiological severity, pulmonary cavity formation, and sputum bacterial load in pulmonary tuberculosis (PTB). For instance, Rolandelli et al. found that individuals carrying the AA genotype of interleukin-17A gene SNP rs2275913 exhibit more severe disease, characterized by marked radiological lung abnormalities and elevated sputum bacterial loads (27). Similarly, Jiang Daobin et al. performed a polymorphism analysis of the IL23R gene in 250 Uyghur PTB patients from Xinjiang, identifying that the TT genotype of rs1884444 was associated with cavity formation and increased lung involvement, thus influencing the severity of PTB (28). Moreover, Najmi et al. studied Toll-like receptor 4 gene polymorphisms among PTB patients in India, demonstrating that carriers of the G allele at rs4986790 had higher sputum bacterial loads, with 30.2% of these patients exhibiting bacterial loads of 2 + and 3+ (29). Given the current paucity of research on host genetic polymorphisms in severe TB, characterized by small sample sizes and limited studies on genetic susceptibility beyond severe PTB, it is crucial to address these research gaps. Therefore, this study aims to comprehensively investigate the impact of host gene polymorphisms on TB severity.

Autophagy is an essential cellular mechanism for regeneration and repair, responsible for removing damaged organelles, denatured proteins, and nucleic acids. This process not only provides defense against pathogen invasion but also protects cells from injury induced by intracellular toxins (11, 30, 31). Autophagy plays a critical role in maintaining cellular homeostasis, combating disease, and delaying aging. ULK1, a serine/threonine kinase, participates in phosphorylation, ubiquitination, and acetylation of autophagy-related proteins (32, 33). It regulates various autophagic and non-canonical signaling pathways and serves as an initiator of autophagy in mammals. Mtb is a major pathogen responsible for human morbidity and mortality; however, certain individuals remain unaffected despite prolonged exposure. This resistance is attributed to Mtb effector molecules modulating human immune responses, enabling the pathogen to evade immune clearance. Autophagy, which restricts intracellular survival of Mtb, represents the most critical host mechanism for limiting pathogen growth. ULK1, involved in regulating autophagic signaling pathways, is therefore crucial in the host defense against TB (34, 35).

Previous research has indicated that, in the Chinese population, carrying the C allele of ULK1 rs7138581 reduces the risk of PTB. Specifically, individuals carrying the CG genotype at rs7138581 exhibit decreased susceptibility to PTB but display more severe clinical parameters following Mtb infection, with 65.9% showing sputum bacterial loads of ++ or +++ (36).

In our research, we identified significant statistical differences in clinical symptoms, laboratory parameters, and other indicators between patients with mild and severe TB. These indicators may serve as risk factors for symptomatic TB. Consequently, it is essential to enhance the identification of clinical risk factors associated with severe TB in clinical settings. Simultaneously, identifying key biomarkers predictive of severe TB is crucial, enabling clinicians to detect high-risk patients early and implement timely preventive interventions. Logistic regression analysis, after adjusting for potential confounding factors, demonstrated that the minor allele G at rs1134574 significantly increased the risk of severe TB (OR^a^ = 23.499, 95% CI: 7.339–75.249, P^a^ < 0.0001). However, no statistically significant differences were observed between severe and mild TB groups regarding genotype frequency distributions or genetic model analyses at this locus. In the clinical phenotype analysis, significant differences among the ULK1 gene rs1134574 AA, AG, and GG genotypes were noted in the prevalence of hot flashes and night sweats (*p* = 0.045) and neutrophil percentage (*p* = 0.046). Furthermore, bioinformatic functional prediction was performed for rs1134574 using RegulomeDB,^1^ an online tool for predicting SNP functions. RegulomeDB analysis assigned rs1134574 a score of 1b, indicating that this SNP likely resides within key regulatory regions (e.g., promoter/enhancer) and potentially influences transcription factor binding or gene expression. The gene location of rs1134574 is presented in Supplementary Figure 1.

Therefore, our research further clarifies clinical differences between mild and severe TB cases, highlighting a genetic susceptibility linking ULK1 gene polymorphisms to severe TB within the Chinese Han population.

However, our study has several limitations. Firstly, TB arises from interactions between genetic and environmental factors. Our analysis did not adjust for certain potential confounders, such as smoking status, treatment history, and drug resistance. Furthermore, strict corrections might increase false-negative findings. Therefore, multiple-test corrections were not applied to all statistical analyses; only age and gender were considered during adjustments for confounders. Secondly, this study was conducted on a Han population aged 15–45 years from Southwest China, which limits the generalizability of our findings to other populations. Finally, further functional validation of rs1134574 is warranted in future studies.

## Data Availability

The datasets presented in this study can be found in online repositories. The names of the repository/repositories and accession number(s) can be found in the article/Supplementary material.

## Footnotes

^1^ http://www.regulomedb.org/
