# Supplementary figures and images for: ULK1 gene polymorphisms and severe tuberculosis in the Chinese Han population: a case-control study
Source: Front Med (Lausanne). 2025 Sep 9;12:1635313. doi: 10.3389/fmed.2025.1635313 (PMC12454327; doi:10.3389/fmed.2025.1635313)

Supplementary Figure 1 The gene location of rs1134574


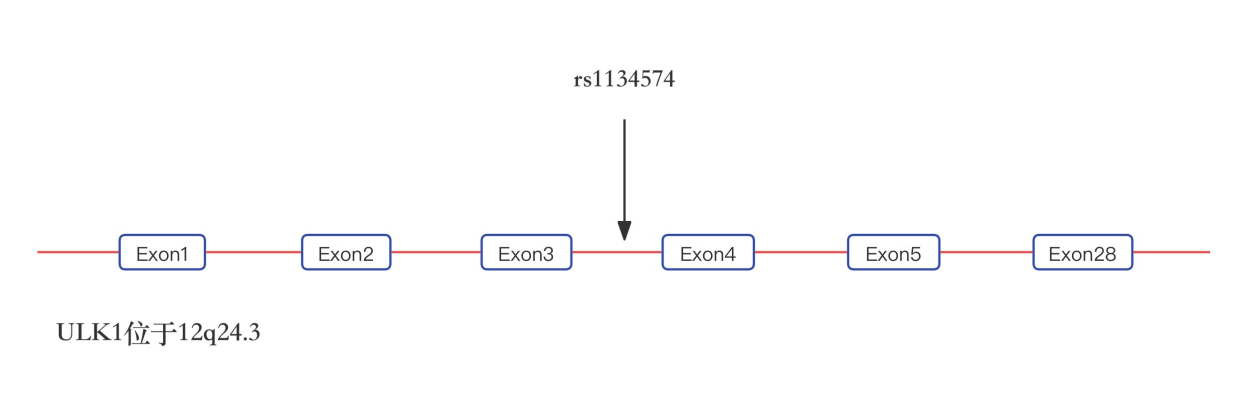

Supplement: Supplementary file 1 [file Data_Sheet_1.DOCX]
